# Autophagy Agents in Clinical Trials for Cancer Therapy: A Brief Review

**DOI:** 10.3390/curroncol29030141

**Published:** 2022-03-05

**Authors:** Samiha Mohsen, Philip T. Sobash, Ghada Fahad Algwaiz, Noor Nasef, Safaa Abed Al-Zeidaneen, Nagla Abdel Karim

**Affiliations:** 1 Faculty of Medicine, University of Toronto, Toronto, ON M5S 1A8, Canada; samiha.mohsen@mail.utoronto.ca; 2 Department of Internal Medicine, White River Health System, Batesville, AR 72501, USA; psobash@wrmc.com; 3 Department of Medicine, King Faisal Specialist Hospital and Research Center, Al Mathar Ash Shamali, Riyadh 11564, Saudi Arabia; ghada_fahad@live.com; 4 College of Arts and Sciences, The Ohio State University, Columbus, OH 72501, USA; noornassef@gmail.com; 5 Department of Allied Medical Science, Al-Balqa’ Applied University, As-Salt 2PF8+XM, Jordan; safaa84@bau.edu.jo; 6 Department of Hematology and Oncology, Medical College of Georgia, Augusta, GA 30912, USA

**Keywords:** autophagy, cancer, hydroxychloroquine, chloroquine, pevonedistat, clinical trial

## Abstract

Autophagy has been of novel interest since it was first demonstrated to have effect in Burkitt’s lymphoma. Since that time, the autophagy agents chloroquine and hydroxychloroquine have become the only FDA (Food and Drug Administration)-approved autophagy inhibitors. While not approved for cancer therapy, there are ongoing clinical trials to evaluate their safety and efficacy. Pevonedistat has emerged as a novel inhibitor through the neddylation pathway and is an autophagy activator. This paper summarizes and presents current clinical trials for hydroxychloroquine (HCQ), chloroquine (CQ), and Pevonedistat for the clinician.

## 1. Introduction

Autophagy is a catabolic process by which the body cells recycle their contents and eliminate unwanted organelles [1]. It was first described by Christian de Duve in 1963 [2]. The process involves identifying and segregating the targeted cellular organelles and directing them toward lysosomes for degradation [3]. It has been recognized through numerous studies that autophagy encompasses a variety of different cellular catabolic pathways. Each pathway targets a distinct category of cellular targets and is tightly regulated by an array of regulatory proteins and enzymes [3,4,5,6].

Macroautophagy is the most widely studied form, making it of interest in modern-day research for clinicians and drug developers [3,7,8,9,10,11]. The process of macroautopaghy is initiated by the formation of double membrane vesicles—autophagosomes—that engulf their target proteins and organelles from the cytoplasm and after maturation fuse with lysosomes for final degradation [12]. Fusion of the late endosomes with plasma membranes is also believed to release the contents as exosomes in the extracellular matrix. Microautophagy is the second pathway. This process is less complex; however, it is equally important. During microautophagy, lysosomes engulf cytoplasmic contents directly for degradation [13]. The final pathway involves chaperone proteins that target the structures meant to be degraded, tag them, and then transport them to lysosomes. There is no membrane sequestration for those targets as in the other two pathways.

Autophagy is a potential therapeutic target for a variety of diseases and was brought to light by results of a study on endemic Burkitt’s lymphoma in Africa, where remission of the disease was linked to wide administration of chloroquine [14]. Chloroquine is an antimalarial agent that was proven to be an autophagy inhibitor [15]. The drug acts by inhibiting the final stages of autophagy by inhibiting fusion of the late endosomes with the lysosomes. This effect results in the accumulation of late endosomes and the redirection of their contents to the extracellular space as exosomes [16].

Among all three forms, macroautophagy has been the focus of clinical research since it has been linked to numerous vital processes such as antigen presentation [17], immunity [18,19,20], drug resistance [21,22,23], exosomes secretion [24,25], apoptosis [26], inflammation [27], and oncogenesis [28]. More recently, researchers have focused on the chronological order of administration of this drug along with other antineoplastic therapy for optimization in combination therapy [29]. The hypothesis of adding these agents is to minimize cancer cell stress adaptive mechanisms and drug resistance. This paper reviews the current FDA-approved autophagy agents chloroquine and hydroxychloroquine, along with a new and novel pro-apoptotic and autophagy inducer, Pevonedistat, in current clinical trials.

## 2. Materials and Methods

In this review, we looked at clinictrials.gov with the terms “cancer” and drug names hydroxychloroquine, chloroquine, and pevonedistat. Trials were excluded that did not involve cancer treatment. Due to the heterogeneity of study type, phase, cancer type, and treatment, the studies were not evaluated cooperatively but summarily.

## 3. Discussion

### 3.1. Historical Context of Autophagy Agents in Cancer

The role of autophagy agents in cancer is somewhat controversial [30]. There are multiple mechanisms of autophagy dysregulation that may be conducive to cancer growth. As stated previously, stress in the microenvironment along with reactive oxygen species (ROS) and ischemia can upregulate autophagocytic genes to promote autophagy. This process is in response to stressors in the milieu. Interestingly enough in cancer cells, despite their presence in these high cellular stress environments, they do not upregulate autophagy. This is due to their basal level of autophagy being considerably elevated relative to normal cells. While the exact mechanism is unclear, there are theorized mechanisms such as oncogenic mutations causing an inhospitable environment leading to autophagy enhancement for overall survival [31]. The disruption and inhibition of this upregulated environment for the cancer cells has become a novel discussion in recent years as a potential target.

### 3.2. Autophagy Pathways

#### 3.2.1. Lysosomal Pathway

The lysosomal pathway in autophagy has been the most widely studied. This process involves lysosomal digestion and recycling cytoplasmic components for cellular recycling. This differs from the contrasting ubiquitin–proteasome system (UPS), which is specifically mediated through the process of degradation of ubiquitinated proteins. Although a complex process, there appears to be crosstalk between the two pathways. While originally thought to be the “garbage disposal” of the cell, it is now known this process plays further roles such as intracellular transport, transduction signaling, and metabolism. The lysosomal pathway is an important potential therapeutic target because of its role in upregulation during cancer formation and growth [32]. Due to the increasingly inhospitable environment with rapidly dividing cells, resources become scarce. Therefore, upregulation of lysosomal degradation and an increase in lysosomal numbers for intracellular components becomes necessary for cellular survival. Lysosomes regulate intra- and extracellular pH, helping ensure adequate homeostasis. Chloroquine and Hydroxychloroquine act through unclear mechanisms that do not allow lysosomes to fuse with autophagosomes while retaining their enzymatic function. Bafilomycin A1, through inhibition of the lysosomal V-ATPase, prevents acidification, causing autophagosomes that are engulfed by lysosomes of the vesicular organelles in its lumen, leading to cell death [16].

#### 3.2.2. Neddylation Pathway

Neddylation has become an increasingly novel focus over the past few years as a target for anti-cancer therapy. This process has been shown to be overactive in a variety of human cancers, thus making it a desirable choice of therapeutic targeting [33]. It is similar to the ubiquitination pathway, with the exception of utilizing the NEDD-8 activating enzyme (NAE) as opposed to the E1 enzyme. The novel therapeutic Pevonedistat (MLN4924) has been shown to significantly increase cell apoptosis and autophagy via neddylation [34]. Neddylation may be an increased therapeutic benefit, as it also has other effects on the tumor microenvironment in comparison with other pathways [33].

### 3.3. Clinical Trial Results

#### 3.3.1. Chloroquine (CQ)

As stated previously, the only FDA-approved autophagy agents are Chloroquine and Hydroxychloroquine [35]. Both function by inhibiting lysosomal acidification. In acidic environments, they become protonated and are then trapped inside the lysosome, causing increasing pH. This increase in pH inhibits the ability of enzyme degradation, therefore creating a cytotoxic effect via the increased pH, as the autophagy mechanism is inhibited. This blocks a survival mechanism that allows cancer cells to proliferate. The variability in effect of these therapies in different cancer lines can vary greatly, partly attributable to differing cancer types relying more on autophagy than others [36]. In trials for Chloroquine, monotherapy slightly outnumbered combination. This is in contrast with Hydroxychloroquine and Pevonedistat, where the majority of studies were conducted on combination therapies. Studies have demonstrated that combination therapy may seemingly work better than monotherapy [37]. The most studied cancer types in chloroquine trials were brain and breast, followed by lung and GI. These studies are outlined in Table 1 and Table 2 and are discussed below.

##### Brain

In a phase I trial with temozolomide, there was a favorable toxicity profile [46]. A phase II trial also demonstrated excellent tolerability in patients receiving whole brain radiation therapy (WBRT) [44]. The only phase III trial showed an enhanced response in glioblastoma multiforme. Chloroquine coadministration with Carmustine enhanced activity in resistant clones during radio and chemotherapy [47].

##### Breast

Chloroquine was associated with positive results in two out of three trials. A phase I/II study demonstrated a measurable reduction in proliferation in DCIS. There was also immune cell migration, specifically with macrophages, into the ducts that was observed [42]. One study with single agent used preoperatively failed to demonstrate any clinically significant effects on proliferation [38]. They concluded that the toxicity was negligible compared with placebo but that the effects were also negligible.

In a phase II randomized control trial (RCT) trial by Arnaout, KI67 index was demonstrated to not be lowered with use of Chloroquine supplementation as a mono therapy compared with placebo. It was speculated that autophagy inhibitors may be more beneficial in combination therapy and more synergistically beneficial when tumor cells are placed under stress [38]. This highlights the overall generality seen, in which combination therapy with antineoplastic agents in coadministration with Chloroquine sees better response, specifically with proliferation, in the clinical trials presented.

#### 3.3.2. Hydroxychloroquine (HCQ)

##### Miscellaneous Tumors

Rosenfeld et al. provided data showing a dose-limiting toxicity with HCQ use with temozolomide. No reduction in proliferation was demonstrated [59]. High-dose HCQ and dose-intense temozolomide were found to be safe and tolerable and were associated with autophagy modulation in patients with melanoma [54].

##### Lung

Modest improvements in clinical responses have been shown in HCQ with carboplatin/paclitaxel/bevacizumab treatment compared with previous studies [49]. Further phase II trials have shown promising effects in Kras-mutated lung cancers, opening up the possibilities of further therapeutic options in chemoresistant cancers [52]. The only other lung cancer trial, phase I, showed a safe toxicity and tolerability profile when given with erlotinib [58].

##### Gastrointestinal

In pancreatic trials, monotherapy demonstrated inconsistent autophagy inhibitory effects along with clinically insignificant therapeutic efficacy [48]. Other monotherapy trials demonstrated safe toxicity profiles but were not clinically significant and showed no difference in outcomes with p53 status of individual patients. These trials contrasted with combination therapy including gemcitabine and nab-paclitaxel, in which greater pathologic and biomarker response increased, along with autophagy inhabitation and immune activity [56].

Colorectal cancer showed fewer promising results, with some findings from phase I and I/II. HCQ, regorafenib and etinostat were poorly tolerated [50]. In another trial comparing vorinostat and HCQ combination with Regorafenib alone, there was no survival improvement [51]. A phase I/II with folfox/bevacizumab with HCQ was well tolerated, although the second phase of study has not compiled results at this time [55].

##### Renal Cell Carcinoma

HCQ with everolimus showed tolerability in combination phase I/II trial. The primary endpoint of a 6-month PFS of 40% was met, showing encouraging results [62]. Another phase I trial with IL-2 (no chemotherapy) also demonstrated a PFS of 17 months with a 600 mg dose monotherapy [64].

##### Multiple Myeloma

Both the multiple myeloma trials concerning HCQ were phase I combination trials, one with bortezomib and cyclophosphamide/rapamycin. The addition of mammalian target of rampiclin (mTOR) and autophagy inhibition with cyclophosphamide yields a regimen with low toxicity that is tolerable in heavily pretreated patients. Both trials stated that phase II trials are needed for further elucidation of synergistic effects with other myeloma regimens [60,61].

### 3.4. Pevonedistat (MLN4924)

A more recent drug that has sparked clinical interest is Pevonedistat, with a more novel pathway of NEDD-9 inhibition leading to autophagy activation. Pevonedistat differs in terms of CQ/HCQ in that it is a pro-apoptotic and autophagy inducer, rather than inhibitor. Many of the clinical trials conducted were for hematological malignancies. Many of these trails were combination therapy vs. monotherapy. There were two phase II trials out of 8 total trials, with one being terminated and one showing promising results. Trial NCT02610777, involving Pevonedistat plus azacytidine vs. azacytidine alone, had promising OS, EFS, ORR, and OS, with similar patient side effect profiles [70]. All other phase I trials demonstrated generally well-tolerated side effects with Pevonedistat use [66,67,68,69,71]. Two trials had unavailable results—one due to termination and one with an inaccessible abstract.

### 3.5. Autophagy Inhibitors as Monotherapy vs. Combination

In cancer cells, multiple studies have demonstrated that autophagy suppression along with creating a cytotoxic environment with chemotherapy can disrupt cancer homeostasis far greater than either agent alone. Combination chemotherapy has been a cornerstone of cancer therapy, and in this regard the data seem to be applicable to autophagy as well. As seen in Table 1, the clinical trials associated with these show a higher number in combination as compared with monotherapy alone.

Theories have been proposed as to why combination may have seemingly more clinically efficacious results—the main theory being oxidation with use from antineoplastic therapy creates intracellular stress, ROS, and invokes starvation creating cytotoxic environments. The upregulated autophagocytic process normally present at a basal level in cancer cells is unable to function properly when antiautophagocytic agents are present [72]. Another is autophagy inhibitors and pro-apoptotic agents’ ability to enhance cancer cell radiosensitivity [72,73]. While not widely studied, autophagy inhibitors and activators (mTOR inhibitors) have been used with synergistic results [74]. This could lead to novel pathways in mTOR-resistant cancers.

### 3.6. Adverse Effects Associated with Autophagy Agents

Due to the recency of which these agents have been utilized in a clinical setting, we cannot fully evaluate the potential long-term side effects. Although these may be used as a tumor suppressor and may aid in malignant therapeutics, considerations must be taken into effect on potentiating other diseases, such neurodegenerative diseases in which autophagy can be protective [75]. A limitation of this review is that the trials are mainly phase I and/or II and that side effects may be present that were not discovered at this stage. However, note that most trials did not indicate that such toxicities were associated with results, and they did not demonstrate harmful effects that would prevent progression to a phase II or III trial. Side effects associated with use were mainly GI related, with nausea, emesis, and diarrhea.

### 3.7. Tumor Response and Ki67

A randomized control trial that included 70 breast cancer patients was designed to evaluate effects on tumor of daily CQ supplementation at a dose of 500 mg daily. It was found that there was no significant difference in tumor Ki-67 index between both CQ and placebo groups [38]. Similarly, a phase II randomized clinical trial found that HCQ combination with gemcitabine and nab-paclitaxel did not improve 12-month overall survival among patients with metastatic pancreatic cancer. However, in the same study, overall response rate of advanced tumors showed improvement with continuous addition of 600 mg HCQ twice daily that may permit resection of tumor. A similar study analyzed the effect of combining HCQ with pre-operative gemcitabine and nab-paclitaxel chemotherapy in patients with pancreatic adenocarcinoma and found significant results. This study employed 64 patients and found administration of HCQ correlated with an increase in the means of patients with the tumor. This immune system alteration in the resected tumor correlated with improved overall survival in recurrence-free survival [56]. It is not surprising given that Pevonedistat, being a newer drug, has clinical trials that are less numerous, and therefore limited results.

### 3.8. Strengths and Weakness of Autophagy Inhibitors in Cancer

While Chloroquine does impact intracellular pH in acidic parts of the cell, it is limited in its ability to permeate the cellular membrane in the presence of an acidic environment [76,77]. This is highly disadvantageous since the tumor microenvironment is more acidic than at homeostatic levels. This can be attributed to the Warburg effect in which cancer cells use glycolysis in lieu of anaerobic respiration [77]. As discussed previously, effects of autophagy inhibitors can inadvertently promote antitumor mechanisms. This mechanism occurs through pathways such as the promotion of T-regs that can damper down the innate immune system alarm for tumor escape and upregulation in transcription factors in conjunction with other anti-neoplastic therapies due to cellular stress. For example, alkylating agents activate ataxia telangiectasis-mutated kinase (ATM), which increases cancer cells upregulation of autophagy to help in survival [78]. In turn, this increases a protective effect in regular glycolysis, resulting in increasing lactate creating the acidic environment that may be detrimental to Chloroquine/Hydroxychloroquine effects. The effect of acidification must be taken into account as well with other therapeutic options.

Depending on cellular conditions, autophagy has been shown to increase and decrease immune response in certain immunotherapies [79]. This can be seen by immunological response of CQ-immune cancer antigens. In immunocompetent mice, CQ decreased acidification into lysosomes, which in turn has been shown to hinder CD4 positive T cells. Conversely, in the T cell deficient, most Chloroquine has been demonstrated to increase NK cells and upregulate TNF, leading to more antitumor response [78]. One potential benefit of autophagy inhibition is the evidence demonstrating the lower risk of metastasis. This mechanism is achieved through the disruption of epithelial to mesenchymal transfer. Of note, this benefit is limited to malignant cells that are less differentiated and possess more stem cell-like features. These limitations have also been seen with mTOR inhibitors’ ability to slow progression but limited cell death [80].

## 4. Conclusions and Future Directions for Autophagy Agents in Cancer Therapy

Both Chloroquine and Hydroxychloroquine demonstrate inhibitory effects on autophagy, making them potential targets for future cancer therapies. In this paper we have provided an overview of the characteristics of the clinical trials with Chloroquine, Hydroxychloroquine, and novel drug Pevonedistat. Interestingly, to our knowledge, there are no current trials combining agents that are pro-apoptotic and autophagy inhibitors. Given that Chloroquine and Hydroxychloroquine are autophagy inhibitors, while Pevonedistat is a pro-apoptotic agent, this has potential to lead to more interesting results used in combination to approach multiple sites of therapeutic significance.

There are limitations to use of autophagy inhibitors, and these can also be seen with mTOR inhibitors, as discussed previously. Further understanding of the process with future clinical trials will likely yield information and results that guide drug discovery. While Chloroquine and Hydroxychloroquine are the only FDA-approved autophagy inhibitors, there are promising results in terms of toxicity in early phase studies, but further clinical trials are needed to see clinical benefit with other therapies.

## Figures and Tables

**Table 1:** Characteristics of clinical trials of Chloroquine, Hydroxychloroquine, and Pevonedistat in clinical trials of cancer treatment by therapy type (monotherapy or combination with chemo), cancer type (liquid or solid), and trial phase.

Therapy Type	Chloroquine	Hydroxychloroquine	Pevonedistat
Monotherapy	11	20	3
Combination	7	66	34
Total	18	86	37
**Cancer Type**			
MDS	-	1	13
Multiple Myeloma	1	4	2
AML	-	2	19
ALL	-	-	1
CLL	-	1	
Lymphoma	-	-	4
Non-Hematological			
Brain	7	4	1
Brain Metastasis	1	-	-
Endocrine	1	1	-
Lung	2	8	2
Breast	3	9	-
GI	2	22	1
Sarcoma/Bone Mets	1	4	-
Melanoma	1	8	1
Solid Neoplasms	1	8	5
Renal	-	1	-
Prostate	-	9	-
Ovarian	-	1	-
Lymphangioleiomyomatosis	-	1	-
Mesothelioma	-	-	1
**Phase**			
Phase I	6	28	19
Phase I/II	3	25	5
Phase II	5	29	10
Phase II/III	-	1	-
Phase III	1	-	2
Not Applicable	3	2	1
Published Results	10	16	8

**Table 2:** Clinical Trials for Chloroquine, Hydroxychloroquine, and Pevonedistat with results. Included are chemotherapy and/or immunotherapy included in trials.

Trial	Indication	Chemotherapy/ Immunotherapy	Phase	Results
**Chloroquine**				
NCT02333890 [38]	Breast	N/A	II	Treatment with single-agent Chloroquine 500 mg daily in the preoperative setting was not associated with any significant effects on breast cancer cellular proliferation. It was, however, associated with toxicity that may affect its broader use in oncology.
NCT01446016 [39]	Breast	Taxane	II	A combination of Chloroquine with taxane or taxane-like chemotherapy was efficacious in patients with locally advanced or metastatic breast cancer with prior anthracycline-based chemotherapy.
NCT02071537 [40]	Advanced Solid Tumors	Carboplatin Gemcitabine	I	Maximum tolerated dose of CQ was lower than previously reported with concomitant use of chemotherapeutic regimens.
NCT01777477 [41]	Pancreatic	Gemcitabine	I	The addition of Chloroquine to gemcitabine was well tolerated and showed promising effects on the clinical response to the anti-cancer chemotherapy. Based on these initial results, the efficacy of the Gemcitabine–Chloroquine combination should be further assessed.
NCT01023477 [42]	Breast	N/A	I/II	Oral Chloroquine, as anti-autophagy therapy, generates a measurable reduction in proliferation of DCIS lesions and enhances immune cell migration into the duct.
NCT02496741 [43]	Solid Tumors (Glioma/ Cholangiocarcinoma/ Chondrosarcoma	N/A	1/II	The combination regimen of metformin and Chloroquine is well tolerated, but the combination did not induce a clinical response in this patient population.
NCT01727531 [44]	Brain Metastasis	N/A	II	WBRT with concurrent, short-course CQ is well tolerated in patients with brain metastases. The high intracranial disease control rate warrants additional study.
NCT01438177 [45]	Multiple Myeloma	Velcade Cyclophosphamide	II	The addition of Chloroquine to bortezomib and cyclophosphamide is effective and overcoming probably some inhibitor resistance in a significant fraction of heavily pre-treated patients, with an acceptable toxicity profile.
NCT02378532 [46]	Brain	Temozolomide	I	A daily dose of 200 mg CQ was established as the MTD when combined with RT and concurrent TMZ for newly diagnosed GBM. Favorable tolerability supports further clinical trials.
NCT00224978 [47]	Brain	Carmustine	III	Chronic administration of Chloroquine greatly enhanced the response of GB to antineoplastic treatment. Because the toxicity of Chloroquine on malignant cells is negligible, these favorable results appear mediated by its strong antimutagenic effect that precludes the appearance of resistant clones during radio and chemotherapy.
**Hydroxychloroquine**				
NCT01273805 [48]	Pancreatic	N/A	II	HCQ monotherapy achieved inconsistent autophagy inhibition and demonstrated negligible therapeutic efficacy.
NCT00765765	Breast	Ixabepilone	I/II	Terminated/No published data.
NCT00786682	Prostate	Docetaxel	II	Terminated/No published data.
NCT01828476	Prostate	Abiraterone ABT-263	II	Terminated/No published data.
NCT01006369	Colorectal	Capecitiabine Oxalplatin Bevacizumab	II	Completed/No published data.
NCT00728845 [49]	Lung	Carboplatin Paclitaxel Bevacizumab	I/II	Addition of Hydroxychloroquine is safe and tolerable, with a modest improvement in clinical responses compared with prior studies. Autophagy inhibition may overcome chemotherapy resistance in advanced NSCLC, and further study in a more molecularly selected population such as KRAS-positive tumors is warranted.
NCT02346340 [50]	Colorectal	Vorinostat Regorafenib	II	VOR/HCQ did not improve survival when compared with RGF.
NCT03215264 [51]	Colorectal	Regorafenib Entinostat	I	The combination of regorafenib, HCQ, and entinostat was poorly tolerated without evident activity in metastatic colorectal cancer.
NCT01649947 [52]	Lung	Carboplatin Paclitaxel Bevacizumab	II	Addition of HCQ has a similar toxicity profile compared with chemotherapy alone. Response rate to therapy in Kras-mutated and wild-type tumors was similar; even the Kras-mutated tumors usually demonstrate worse responses and progression free survival to chemotherapy.
NCT02232243 [53]	Prostate/Lung/Head and Neck	N/A	I	Both dose levels of HCQ were well tolerated, and Par-4 secretion but not induction of the autophagy-inhibition of marker p62 correlated with apoptosis induction in tumors.
NCT00714181 [54]	Solid Tumors/Melanoma	Temozolomide	I	This study indicates that the combination of high-dose HCQ and dose-intense TMZ is safe and tolerable and is associated with autophagy modulation in patients.
NCT01206530 [55]	Colorectal	FOLFOX Bevacizumab	I/II	The combination of FOLFOX/bevacizumab with HCQ is an active regimen in unselected patients with colorectal cancer. A randomized phase II trial of the combination is in development.
NCT01128296 [56]	Pancreatic	Gemcitabine	I/II	Pre-operative autophagy inhibition with HCQ plus gemcitabine is safe and well tolerated. Surrogate biomarker responses (CA19-9) and surgical oncologic outcomes were encouraging. p53 status was not associated with adverse outcomes.
NCT01506973 [57]	Pancreatic	Gemcitabine/nab-Paclitaxel	I/II	The addition of HCQ to block autophagy did not improve the primary endpoint of overall survival at 12 months. However, improvement seen in the overall response rate with HCQ may indicate a role for HCQ in the locally advanced setting, where tumor response may permit resection.
NCT 00977470 [58]	Lung	Erlotinib	I	HCQ with or without erlotinib was safe and well tolerated. The recommended phase 2 dose of HCQ was 1000 mg when given in combination with erlotinib 150 mg.
NCT00486603 [59]	Brain	Temozolomide	I/II	These data establish that autophagy inhibition is achievable with HCQ, but dose-limiting toxicity prevented escalation to higher doses of HCQ. Patients are awaiting the development of lower-toxicity compounds that can achieve more consistent inhibition of autophagy than HCQ.
NCT00568880 [60]	Multiple Myeloma	Bortezomib	I	Combined targeting of proteasomal and autophagic protein degradation using bortezomib and Hydroxychloroquine is therefore feasible and a potentially useful strategy for improving outcomes in myeloma therapy.
NCT01396200 [61]	Multiple Myeloma	Cyclophosphamide Rapamycin	I	The addition of mTOR and autophagy inhibition to a backbone of cy/dex yields a tolerable regimen with durable responses in heavily pretreated patients. A randomized phase 2 study is needed to determine the synergistic properties of dual mTOR and autophagy inhibition vs. chemotherapy alone.
NCT01550367 [62]	RCC	N/A (IL-2)	I/II	IL-2 plus HCQ was well tolerated and clinically active, with encouraging PFS of 17 months at the 600 mg HCQ dose.
NCT01687179 [63]	Lymphangioleiomyomatosis	Sirolimus	I	The combination of Sirolimus and Gydroxychloroquine is well tolerated, with no dose-limiting adverse events observed at 200 mg twice a day. Potential effects on lung function should be explored in larger trials.
NCT01510119 [64]	RCC	Everolimus	I/II	Combined Hydroxychloroquine 600 mg twice daily with 10 mg daily everolimus was tolerable. The primary endpoint of >40% 6-month PFS rate was met. Hydroxychloroquine is a tolerable autophagy inhibitor for future RCC or other trials.
NCT03344172	Pancreatic	Gemcitabine, Abraxane, Avelumab	II	Terminated/No published results.
NCT01978184 [65]	Pancreatic	Gemcitabine, Abraxane	II	The addition of Hydroxychloroquine to preoperative gemcitabine and nab-paclitaxel chemotherapy in patients with respectable pancreatic adenocarcinoma resulted in greater pathologic tumor response, improved serum biomarker response, and evidence of autophagy inhibition and immune activity
NCT02013778	HCC	N/A (TACE)	I/II	Terminated/No published data.
**Pevonedistat**				
NCT01862328 [66]	Solid Tumors	Paclitaxel Gemcitabine Docetaxel Carboplatin	I	Pevonedistat with docetaxel or with carboplatin plus paclitaxel was tolerable without cumulative toxicity. Sustained clinical responses were observed in pretreated patients receiving Ppevonedistat with carboplatin and paclitaxel.
NCT03057366 [67]	Solid Tumors	Docetaxel Carboplatin Paclitaxel	I	Pevonedistat in combination with docetaxel or carboplatin plus paclitaxel was generally well tolerated.
NCT01814826 [68]	AML	Azacitidine	I	Pevonedistat/azacitidine combo did not alter toxicity profile of azacytidine. Intent to treat ORR was 50%.
NCT00722488	Multiple Myeloma Lymphoma	N/A	I	Unable to obtain abstract.
NCT03709576	AML	Azacitidine	II	Terminated/No published results.
NCT01011530 [69]	Melanoma	N/A	I	MLN4924 is generally well tolerated at the doses tested on schedule A, with antitumor activity.
NCT02610777 [70]	MDS	Azacizidine	II	The OS, EFS, and ORR benefits were particularly promising among patients with higher-risk MDS, as was the OS benefit in LB-AML. The addition of Pevonedistat to azacitidine resulted in a comparable safety profile to azacitidine alone, no increased myelosuppression, and azacitidine dose intensity was maintained.
NCT00911066 [71]	AML MDS	N/A	1	Administration of the first-in-class agent, Ppevonedistat, was feasible in patients with MDS and AML, and modest clinical activity was observed.
